# *Campylobacter* Biofilms: Potential of Natural Compounds to Disrupt *Campylobacter jejuni* Transmission

**DOI:** 10.3390/ijms222212159

**Published:** 2021-11-10

**Authors:** Bassam A. Elgamoudi, Victoria Korolik

**Affiliations:** 1 Institute for Glycomics, Griffith University, Gold Coast, QLD 4222, Australia; b.elgamoudi@griffith.edu.au; 2 School of Pharmacy and Medical Science, Griffith University, Gold Coast, QLD 4222, Australia

**Keywords:** *Campylobacter*, biofilm, natural compounds, antibiofilm

## Abstract

Microbial biofilms occur naturally in many environmental niches and can be a significant reservoir of infectious microbes in zoonotically transmitted diseases such as that caused by *Campylobacter jejuni*, the leading cause of acute human bacterial gastroenteritis world-wide. The greatest challenge in reducing the disease caused by this organism is reducing transmission of *C. jejuni* to humans from poultry via the food chain. Biofilms enhance the stress tolerance and antimicrobial resistance of the microorganisms they harbor and are considered to play a crucial role for *Campylobacter* spp. survival and transmission to humans. Unconventional approaches to control biofilms and to improve the efficacy of currently used antibiotics are urgently needed. This review summarizes the use plant- and microorganism-derived antimicrobial and antibiofilm compounds such as essential oils, antimicrobial peptides (AMPs), polyphenolic extracts, algae extracts, probiotic-derived factors, d-amino acids (DAs) and glycolipid biosurfactants with potential to control biofilms formed by *Campylobacter*, and the suggested mechanisms of their action. Further investigation and use of such natural compounds could improve preventative and remedial strategies aimed to limit the transmission of campylobacters and other human pathogens via the food chain.

## 1. Introduction

Bacteria typically prefer to grow in biofilms and complex communities where they are protected from physical trauma, host immune responses, desiccation and antimicrobial agents [1,2,3,4,5]. In this mode, bacteria exude gelatinous exopolymeric substances that are mostly polysaccharides, proteins and DNA. Progressively, a structured biofilm matrix or a gel, containing bacteria and exuded elements, is formed. In nature, bacterial biofilms almost always consist of multiple microbial species, and are readily formed on both biotic and abiotic surfaces such as tissues, medical devices and prostheses.

*Campylobacter* *jejuni,* and its close relative *Campylobacter coli,* are capable of forming mono and multi-species biofilms [6], and are the most common foodborne bacterial pathogens. They are the cause of annual diarrhoeal disease for about 10% of the world’s population (WHO) including 200 million children, resulting in human suffering and high economic burden [7,8]. The clinical features of *C. jejuni* gastroenteritis range from mild, non-inflammatory diarrhoea to severe abdominal cramps and febrile bloody diarrhoea that requires hospitalisation and antimicrobial chemotherapy. *C. jejuni* can also cause post-infection complications, including those associated with acquired immune-mediated neuropathies of the peripheral nervous system such as Guillian Barré Syndrome (GBS), resulting in neuromuscular paralysis [9]. Other complications such as meningitis, urinary tract infections and bacteraemia have also been reported [10,11].

These *Campylobacter* spp. can be found in water reservoirs, as commensals in the intestinal tract of animals, particularly birds, and as virulent pathogens in humans. The animal reservoirs play an important role in transmission of infectious organisms to humans and include domestic and wild animals [12,13,14,15]. Contaminated animal food products, poultry, in particular, are considered to be a major source of bacteria causing human campylobacteriosis [10,16]. *Campylobacter* spp. are also able to survive well in the open-air farm environment and can be isolated from unpasteurized milk, raw vegetables, soil and surface water [13,17,18]. Several potential survival mechanisms of *Campylobacter* spp. have been suggested, such as stationary phase survival mechanism, stress responses (i.e., thermal stress response), viable but nonculturable state (VBNC), and, of course, biofilm formation [19,20,21,22,23]. Biofilms have been implicated in transmission of campylobacter disease via complex mixed-species communities that form part of the natural microbiota in chicken caeca and animal intestines. Campylobacteria then persist in surface-type biofilms on animal food products and packaging [6,14,24,25]. Therefore, similar to other bacterial pathogens, the ability to form biofilms is an important virulence mechanism in relation to transmission of disease causing campylobacteria to humans [1,26,27].

## 2. *Campylobacter* spp. Biofilm Formation and Regulation

The formation of biofilms significantly increases the ability of *C*. *jejuni* to survive in extreme conditions [28,29]. For instant, biofilm encased campylobacter cells survive twice as long under atmospheric conditions, and had been shown to form strong biofilms under aerobic condition [15,30]. Biofilm formation is also recognized as a potential reservoir for antimicrobial resistance and is known to facilitate exchange of resistance genes between pathogenic and commensal bacteria [31]. This is particularly pertinent in case of *Campylobacter* spp., including *C. jejuni* and *C. coli*, which exhibit intrinsic resistance to many antimicrobial agents and are naturally conjugative [32,33,34]. In addition, *Campylobacter* spp. are becoming increasingly resistant to the most frequently prescribed antibiotics such as erythromycin, tetracycline and fluoroquinolones, and have been listed by WHO as a priority pathogen for the development of new antibiotics [35,36]. The usage of antibiotics in food animals to control, prevent and treat infections, and to enhance growth, has been implicated in an increased resistance to multiple antibiotics by *Campylobacter* spp. [37]. Majority of *C. jejuni* and *C. coli* are now resistant to at least one of the currently used antibiotics, such as penicillin, trimethoprim, sulfamethoxazole, rifampicin and vancomycin [37], requiring alternative treatments with either gentamicin or third-generation cephalosporins [38].

Several studies have shown that *C. jejuni* strains are able to attach to, and form mono- or mixed-species biofilms with other bacterial species such as *Pseudomonas aeruginosa*, *Escherichia coli*, *Staphylococcus simulans*, *Enterococcus faecalis,* *Salmonella* spp., *Flavobacterium* spp., and *Corynebacterium* spp. [6,39,40]. The evidence from these recent publications suggests that the composition of *Campylobacter* spp. biofilms is similar to that formed by other organisms. While there has been some investigation of the extracellular matrix components of *C. jejuni* biofilms, the architecture and the composition of these are yet to be fully characterized. *C. jejuni* NCTC strain 11168 was reported to produce an extracellular fibre-like material as a component of its biofilm, structurally resembling a net-like matrix [8]. Such matrices contribute to biofilm-mediated antimicrobial resistance, either by acting as a diffusion barrier or by binding directly to antimicrobial agents and preventing their access to the biofilm-encased cells [26]. The extracellular DNA (eDNA) is important for establishment and maintenance of *C. jejuni* biofilm [41,42], and appears to be a crucial component of the extracellular matrix of mature biofilms as degradation of eDNA results in reduction of biofilm formation by *C. jejuni* [41,42,43]. Interestingly, Gaasbeek et al. [44] found that a *C. jejuni* Mu-like prophage-integrated element 1 (CJIE1) containing strain, a non-naturally transformable strain, has a gene encoding an extracellular DNase (eDNase, CJE0256), and eDNase activity could be detected. It is interesting to note that no eDNase activity could be found in naturally transformable *C. jejuni* strains such as NCTC11168 and 81116.

**Figure 1:**
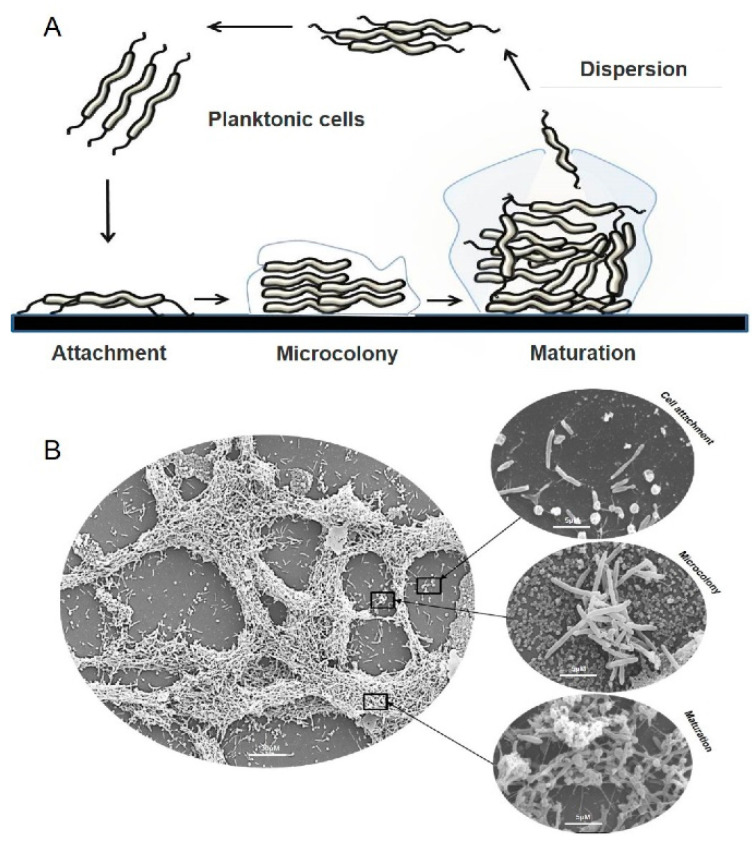
Cycle of biofilm development. (**A**) Planktonic cells swim and attach to surfaces (cell-to-surface and cell-to-cell) resulting in the formation of microcolonies. Mature biofilms can return to a planktonic lifestyle through dispersion and released seed cells complete the cycle of biofilm development. (**B**) Representative scanning electron microscopy (SEM) images of *C*. *jejuni* cultured under microaerobic conditions.

Most of our current knowledge of *Campylobacter* spp. biofilm architecture is summarised in Figure 1. In the first stage of biofilm formation, planktonic cells attach to the surface via two types of interaction: cell-surface and cell-cell interactions using flagella, fimbriae, amyloid-like fibrils and outer membrane proteins [45,46,47]. This process is critical for bacterial adhesion and is influenced by the properties of both bacterial cells and the surface [48,49]. Secondly, after initial attachment, the cells start production of extracellular polymeric substance (EPS) consisting of polysaccharides, extracellular DNA (eDNA) [42], proteins [50], lipids and other glycosylated polymers, in order to initiate micro-colonies and progress to the third stage of a mature biofilm [51,52]. In a mature biofilm, EPS acts as an adhesive between cells and supports the intricate three-dimensional (3D) structure of the biofilm, protecting the cells from toxic compounds such as antibiotics, but allowing the movement of fluid and nutrients [53]. Finally, cell death and autolysis serve as a trigger for the mature biofilm to detach and release cells into the environmental niche in a process called dispersion [54]. Biofilm dispersion is believed to be crucial for the propagation and self-renewal of bacterial communities [53,55] and contributes to bacterial survival, pathogenicity and most importantly, disease transmission [53,56,57].

The understanding of gene regulation of *C. jejuni* biofilm formation is still limited. There are a number of genes known to be involved in the biofilm formation process and include those responsible for motility and chemotaxis [58,59,60], lipooligosaccharide biosynthesis [58,59,61,62], *N*-linked protein glycosylation, capsular polysaccharides (CPS) [58,62,63], and stress response proteins. Quorum sensing (QS), which allows the bacteria to regulate population cell density in biofilms was also found to play a role in *Campylobacter* biofilm formation and to contribute to host colonisation [40,60,64,65]. However, an important messenger, the intercellular bis-(3′-5′)-cyclic dimeric guanosine monophosphate (c-di-GMP), which plays an essential role in the transition between sessile and motile lifestyles in many other organisms [66], or its homologue, is yet to be found in the *C. jejuni* genome.

## 3. Natural Antibiofilm Compounds

Biofilm-disrupting and antimicrobial properties of many naturally occurring compounds against pathogens have been previously explored [67,68,69]. Such compounds (Table 1) include different plant extracts and their components (e.g., containing polyphenols), essential oils (e.g., containing carvacrol) and marine inhabitants (algae extracts), and a number of these have been tested against campylobacters.

### 3.1. Plant-Derived Compounds

Essential oils (EOs) derived from plants are promising antimicrobial compounds, with over ~300 commercially available EOs. Many EOs (e.g., cinnamon oil, clove oil and lavender essential oil) exhibit antibacterial, antibiofilm and antifungal properties which have a wide range of applications in the food and dietary supplement industry [91,92,93,94,95]. EOs are also reported to prevent biofilm formation on abiotic surfaces, which has encouraged the development of alternative disinfection strategies, targeting contaminated surfaces and equipment used in food processing [96,97,98,99]. Moreover, EOs have been added to animal feed and water as taste enhancers for livestock nutrients and as growth promoters, particularly in poultry and porcine farming [94,100,101]. Here, we describe some compounds that exhibit promising antimicrobial and antibiofilm activities against campylobacters.

Cinnamon oil (*Cinnamomum cassia*) and clove oil (*Eugenia caryophyllus*) are reported to have bioactive compounds such as cinnamaldehyde (CA), eugenol (EG) and carvacrol (CR) [92]. These compounds act as antimicrobial and antibiofilm agents against many pathogens including *P*. *aeruginosa*, *Salmonella* Typhimurium, *Streptococcus mutans* and *Listeria monocytogenes* [102,103,104,105]. CA, EG and CR also exhibit an ability to significantly decrease *Campylobacter* spp. biofilms and remove the biofilms from stainless steel and polystyrene surfaces [71,72,73,74,106]. Several studies revealed the effectiveness of CR to reduce *C. jejuni* in vitro and in vivo [107,108,109,110,111,112]. For instance, Wagle et al. [106] found that the minimum inhibitory concentration (MIC) of CR (at 0.002%) was able to reduce the *C. jejuni* adhesion to primary chicken enterocytes (in an in vitro model of chicken intestinal physiology) up to 1.5 log cfu/mL as compared with control. Interestingly, CR downregulated the expression of *C. jejuni* colonisation factors, critical for persistence in the chicken gut, such as chemotaxis (aspartate chemoreceptor, *CcaA*), interactions with host cells (*aspA)* and anaerobic respiration (*NapB*). Similar to that, šimunović et al. [112] demonstrated that CR (MIC 0.0032%), as a pure compound or in synergistic combinations with thymoquinone, and rosmarinic acid, not only has antimicrobial activity against *C. jejuni* but also can increase the antibiotic susceptibility of *C.* *jejuni* by inhibiting the efflux pump activity. Unfortunately, further attempts to determine antibacterial properties of CR against *C. jejuni* using the broiler chicken model were inconsistent. Arsi et al. [113] reported that CR supplemented feed at 0.5–1% could significantly reduce *Campylobacter* counts in broiler chicks, either alone or in combination with thymol. However, their results could not be replicated in other trials, reportedly due to absorption of those compounds before they reach their target, the small intestine and caeca of chickens, or effects of other enteric microflora [109]. To improve the in vivo outcomes, Allaoua et al. [109] used a CR-based product, solid galenic CR formulation, designed to delay the CR release to allow it to reach the caeca of broiler chickens in order to control *C. jejuni*. This new formulation was aimed to preserve the antibacterial efficacy of CR against *C. jejuni* by allowing CR to reach the caeca and large intestine at an effective concentration (at MIC 0.02 mg/mL), which significantly decreased the *C. jejuni* caecal load (by 1.5 log). Kelly et al. [108] also reported that CR was able to reduce *Campylobacter* cell adhesion and invasion of chicken intestinal primary cells and also biofilm formation in vitro. They also showed that CR was able to delay colonisation of chicken broilers by inducing changes in gut microflora. *Campylobacter* spp. was only detected at 35 days of life in the treatment groups compared with the control group where the colonisation occurred at 21 days. Reducing the number of campylobacteria in the chicken intestine is a goal of most studies as quantitative risk assessment models indicate that a reduction of *C. jejuni* numbers on a broiler carcass by 100-fold (or 2 log units) could result in a significant reduction, by 30 times, in the incidence of campylobacteriosis [114]. Even a relatively small reduction in *C*. *jejuni* numbers in the chicken cecum by 1 log_10_ CFU can reduce the public health risk by more than 50% [8]. In addition, CR had a significant effect on *E. coli* numbers in the cecum of the chickens in treatment groups. Similarly, Szott et al. [111] found that CR additive could reduce *C. jejuni* counts in vivo by 1.17 log (up to 28 days of age); however, CR did not successfully reduce *Campylobacter* caecal colonisation in 33-day-old broilers. Interestingly, addition of CR to the diet decreased feed intake increased feed conversion rates and body weight at all levels of supplementation [115]. Similarly, combining basic diet with cinnamon oil (0.3 g of cinnamon oil per kg) could enhance daily weight gain of broiler chickens by 5.1% [116]. One more potential advantage of using CR is its effect on probiotic bacteria where the additional proliferation of probiotic bacteria such as *Lactobacillus* and *Bifidobacteria* spp. has been proposed to be a potential mechanism of inhibiting avian colonisation by disease-causing organisms such as *Campylobacter* spp. [91,117]. The important benefit, all studies agree, is that CR is safe to use as a dietary supplement in the chicken diet and could improve poultry health, feed efficiency, and delay *Campylobacter* colonisation in chickens.

Lavender essential oil (LEO) has antiviral activity against *Herpes simplex* virus type 1 [118]; antibacterial activity against piperacillin-resistant *E. coli* J53 R1, chloramphenicol-resistant *L. monocytogenes* L120, *S. aureus* MRSA and *P. aeruginosa* [119,120,121,122]; and antifungal activity against *Aspergillus niger* and *Aspergillus tubingensis* [123]. LEOs also show an antibiofilm activity against *C. jejuni* with MIC ranged from 0.2 mg/mL to 1 mg/mL [124]. LEOs were reported to downregulate a range of genes (i.e., *Cj0719c*, *kpsS*, *lgt, maf4*, *waaC* and *Cj1467*), involved in the initial attachment of *Campylobacter* spp. cells to abiotic and biotic surfaces. Adaszynska et al. [122] have evaluated the effect of LEO on chicken production by adding LEO to drinking water given to broiler chickens. The results of the experiments not only showed a significant inhibition of microbial growth, but also a significant increase in the body weight of the chickens in the groups receiving LEO as compared with the control group. Similarly, juniper essential oil (JEO) had shown potent anti-adherent effects against *C. jejuni* [67,74,76,125], where flavonoid-rich fractions from juniper, at 1 mg/mL, were able to inhibit attachment of *C. jejuni* cells to polystyrene by up to 70–99%, and reduced the invasion of INT407 cells by 76%. α- and β-pinene are another example of essential oil components from *Alpinia katsumadai* seeds that can have antimicrobial, antimalarial, and antioxidant effects [77,126,127,128]. The antimicrobial activities of (-)-α-pinene were reported against *Campylobacter* spp. in vitro; however, (-)-α-pinene alone showed a low efficacy with MIC_50_ > 500 mg/L required to inhibit 50% of the strains, but when (-)-α-pinene was combined with antibiotics ciprofloxacin and erythromycin, strong potentiating effects against different *Campylobacter* strains were observed. The concentrations of antibiotics could be decreased from 1 mg/mL to 0.002 mg/mL for ciprofloxacin, and from 512 mg/mL to <1 mg/mL for erythromycin [129]. Possible applications of such natural compounds could be in food packaging to maintain food quality and reduce cross-contamination, or as feed additives to increase weight gain of chickens and by reducing the costs associated with antimicrobial feed additives.

Citrus Extracts (CE) have been widely used in many applications in pharmaceuticals and food industry due to their properties as antimicrobial, insecticidal and antifungal agents [130,131]. CEs showed the ability to reduce the biofilm formation of pathogenic bacteria, for example, *Staphylococcus* spp., *Pseudomonas* spp. and *E. coli* due to their antimicrobial activity [132,133,134]. Castillo et al. [79] found that treatment with CE (such as citron, bitter orange, lime, lemon and tangerine) elicits a strong inhibitory effect, up to 75 %, on *C*. *jejuni* biofilm formation. This compound could also decrease the activity of *C. jejuni* quorum-sensing signalling (AI-2 QS) [79]. As another example, grapefruit seed extract (GSE), widely used in the food industry as a safe and effective preservative [135], has an antibiofilm and antibacterial activity against methicillin-resistant *S.* *aureus* (MRSA), vancomycin-resistant *S.* *aureus* (VRSA) and *E. coli* [135,136,137]. GSE can also inhibit *C. jejuni* growth and its adhesion to abiotic and biotic surfaces (at a minimum bactericidal concentration (MBC) of 60 mg/L) [78,138]. GSE consists of many phenolic compounds such as anthocyanins, catechins, flavonols, phenolic acids and proanthocyanidins. Among them, phenolic acids, catechins and proanthocyanidins have a strong inhibitory effects on *C. jejuni* growth [78]. Phenolic acids and catechins could also inhibit the growth of *Campylobacter* strains, with MIC range between 10 and 100 mg/L, which could be useful for the control of *Campylobacter* transmission through the foods chain. In addition, dietary supplements that contain grape seed as a source of phenolic compounds, have shown to promote higher body weight gain in broilers [139]. Further advantage of GSE phenolic compounds is offered by their effectiveness against Gram-positive bacteria via inhibition of the cell wall biosynthesis, and Gram-negative bacteria via break-down of the outer membranes [135,138,140,141]. Currently, GSE is used commercially as a dietary supplement and, therefore, has the potential to be safely used at different points of the food chain to reduce the transmission of campylobacteriosis.

Ethanol solution extract (EREE)*:* Plant-based ethanol extracts have been previously used to control food-borne pathogens and multidrug-resistant bacteria [142,143,144]. For example, *Euodia ruticarpa* ethanol solution extract (EREE) contains bioactive components, such as evodiamine, rutaecarpine and evocarpine which have shown promising antimicrobial activities against *S. aureus* MRSA, mycobacterial strains, and *C*. *jejuni* which are able to inhibit cell adhesion and biofilm formation [80,144,145,146]. EREE exhibited antibiofilm and anti-AI-2 QS properties against *C*. *jejuni* at MIC from 64 to 1024 µg/mL [80], indicating that quinolinone alkaloids have potential to reduce the cell-surface bacterial attachment by interfering with the QS system.

Polyphenolic extracts: Similar to other natural products, polyphenols extracted from plants have been reported to have antimicrobial and antibiofilm activities [68,147,148,149,150,151]. For example, polyphenol-rich cranberry and other berry extracts have strong antibiofilm effect on dual-species *Streptococcus mutans-Candida albicans* biofilms and sole *Streptococcus mutans* biofilms [152,153]. Similarly, polyphenolic components found in spray-dried olive mill wastewater (OMWW-SD) inhibit *Campylobacter* spp. biofilm formation and promote biofilm dispersion [84]. Those polyphenols, mainly secoiridoid and hydroxycinnamic acid derivatives with MIC ranged between 0.15 to 0.3 mg/mL, were able to inhibit biofilm formation by *Campylobacter* strains between 50–92%, depending on concentration. In addition, gallic acid and taxifolin significantly affected CmeABC multidrug efflux pump expression resulting in increased bacterial susceptibility to ciprofloxacin and erythromycin in *C. jejuni* isolates, where 8 μg/mL of the phenolic compounds combined with ciprofloxacin and erythromycin reduced the MIC of those antibiotics 4–32-fold [154]. Green tea is also rich in naturally occurring polyphenolics such as epicatechin (EC), gallocatechin (GC), gallocatechin gallate (GCG), epigallocatechin (EGC), epicatechin gallate (ECG) and epigallocatechin gallate (EGCG). These compounds have potent antioxidant activity and antimicrobial properties [155,156,157]. The extracted EGCG exhibits antimicrobial activity and anti-AI-2 QS activity against *E. coli* [134], and inhibited *C. jejuni* biofilm formation by 75% at concentrations of 31 to 125 μg/mL [83]. This study suggested that green tea extract could be used to restrict growth of *C. jejuni* by interfering with biofilm formation and QS activity, as well as facilitate the performance and health of broilers [158,159].

Another compound to be considered is a commonly used dietary supplement, resveratrol (3,5,4′-trihydroxystilbene). It is produced by several plants and can be found in skin of blueberries and grapes. Resveratrol has been reported to inhibit biofilm formation and to disperse established biofilms and also has an inhibitory activity against a range of bacterial pathogens [85,160]. The antibiofilm activity of resveratrol, with up to 94% *C. jejuni*, and *C. coli* biofilm inhibition at MIC of 0.1–0.2 mg/mL, suggests a potential use of this compound as antibiofilm agent in poultry meat processing, food preparation and packaging. Together, these findings suggest that the use of polyphenolic extracts could be used to limit campylobacterial growth and biofilm formation in animal food products processing, particularly poultry, and consequently enhance food safety and limit the use of chemical additives or preservatives.

Organosulfur compounds: Organosulfur compounds derived from garlic (*Allium sativum*) such as allicin, ajoenes and diallyl sulphide, have shown antimicrobial activity against a vast range of pathogens [161,162,163]. These compounds also have been tested as antimicrobial wash for poultry meat to reduce the number of *C. jejuni* cells [81,82,163]. Organosulfur compounds could be a safer and cheaper alternative to commonly used antimicrobials, such as peracetic acid (PAA), in an effort to reduce contamination during pre- and post-chill carcass and broiler parts treatments [164]. Remarkably, diallyl sulphide was not only able to destroy the EPS structure of the *C. jejuni* biofilm but also eliminated planktonic and sessile cells [82]. Diallyl sulphide, and other bioactive organosulfur compounds, have potential for reducing bacterial cell adherence, inhibiting production of AI-2 QS molecule, and enhancing disruption of cell surface structure of this pathogen. Wagle et al. [81] showed that the application of organosulfur compounds such as diallyl sulphide as antimicrobial wash in postharvest poultry could significantly reduce *C. jejuni* numbers on poultry meat.

Antimicrobial peptides (AMPs) are naturally occurring peptides produced by many multicellular organisms as a first-line immune defence. Many AMPs exhibit broad-spectrum antimicrobial activity which can target both Gram-positive and -negative bacteria [165,166,167,168]. Wheat proteins puroindolines, present in *Triticum aestivum* endosperm, are found in two major isoforms, puroindoline A (PinA) and puroindoline B (PinB). Both have antimicrobial properties due to presence of tryptophan-rich domains (TRDs) [168,169]. TRD-rich peptides have a high affinity for the negatively charged lipids in the bacterial membranes and have antimicrobial effect against many pathogens such as *E. coli*, *S. aureus*, *L. monocytogenes*, and *Aspergillus flavus* [169,170]. The mode of action of PinA is via membrane destabilization, while PinB targets DNA by inhibiting DNA replication [171,172]. In case of *Campylobacter* strains, PinA has been shown to affect both, bacterial growth and biofilm formation [168]. PinA could inhibit *C. jejuni* 81–176 biofilm formation at the concertation of 512 μg/mL and growth at 16–32 μg/mL. Interestingly, using PinA in combination with erythromycin and ciprofloxacin, antibiotics commonly used to treat *C. jejuni* infections, was more effective in reducing *C. jejuni* growth than using any antibiotic alone, indicating a potential use for PinA as an enhancer of antibiotic efficacy.

### 3.2. Microorganism-Derived Compounds

Algae extracts: Many antibacterial compounds have been identified in marine organisms including algal classes such as the *Bacillariophyceae* (diatoms), *Chlorophyceae*, *Chrysophyceae*, *Rhodophyceae* and *Phaeophyceae* [173,174,175]. Algal extracts containing bioactive compounds such as fatty acids and furanone [176,177,178], have been widely used for pharmaceutical and industrial applications. For example, the long-chain fatty acids in the green microalga *Planktochlorella nurekis* has been reported to have antibacterial activity against many pathogens, including *C.* *jejuni,* at concentrations between 0.75–6 mg/mL [179]. Brominated furanone is a naturally occurring polyphenolic compound with antimicrobial properties that can be extracted from *Delisea pulchra* algae. This compound can exert antibiofilm activity against *C. jejuni*, by interfering with AI-2 QS, at MBC 230 μg/mL [83,175]. Such algae have been previously used as safe food additives for poultry [180], as their rich nutrients enhance growth performance and product quality with a possible additional benefit of reducing human food-borne illness.

d-amino acids (DAs): While most proteins are composed of l-amino acids, d-amino acids, DAs, can be found in cell walls of many bacteria. Interestingly, addition of external DAs had been shown to have antibiofilm properties in a variety of species such as *B. subtilis, S. aureus* and *P. aeruginosa* [181,182,183,184]. Moreover, the use of DAs in various combinations enhanced the activity of antimicrobial agents such as colistin, ciprofloxacin and rifampin, frequently used to treat *P. aeruginosa* and *S. aureus* [185]. For *C. jejuni*, a mixture of d-serine, d-Tryptophane and d-methionine at concentration 5 mM was found to be able to inhibit the biofilm formation or disrupt mature biofilm. Moreover, DAs disrupted the ability of *C. jejuni* to form biofilm (up to 70%) by incorporating into peptidoglycan and inducing the disassembly of matrix-associated amyloid fibrils, or by a breakdown of EPS that surrounds the biofilm. DAs were also able to enhance the efficacy of d-Cycloserine (DCS) against *C. jejuni* by up to 32% [47]. DAs appear to be promising antibiofilm compounds and should be further investigated.

Probiotic-derived factors: Several studies have reported the ability of probiotic organisms, such as *Lactobacillus* spp., to secrete probiotic factors (e.g., bacteriocin and reuterin) which have shown antimicrobial activities against various enteric pathogens such as *E. coli,* and *Vibrio cholerae* [186]. In addition, these factors have also been reported to have a beneficial effects on the intestinal epithelium through an improvement of intestinal barrier function leading to reduced permeability to pathogens [187]. For example, bacteriocins of probiotic *Lactobacilli*, are naturally produced as secondary metabolites, display antimicrobial, antiviral and antifungal activities. Bacteriocins have been recognized as non-toxic alterative antibiotics against gastrointestinal infections [188,189]. Bacteriocins had also shown antibiofilm activity against *P. aeruginosa* PAO1 and *B. subtilis* BM19. Bacteriocins act by interfering with the membrane integrity of bacterial cells leading to cell death [186]. Several bacteriocins have been isolated and characterized from commensal bacteria in chicken intestines, such as *Enterococcus faeciu* (E50-52) and *Lactobacillus salivarius* (e.g., OR-7) and can inhibit proliferation of *Campylobacter* spp. [90,188,190]. For instance, feeding bacteriocins E50-52, MIC ranged from 0.025 to 32 µg/mL, to broiler chickens reduced *C. jejuni* cell numbers by more than 99% in the caeca [90].

Another example of probiotic-produced antimicrobial compound is reuterin, which is produced as a byproduct of anaerobic fermentation of glycerol by *Lactobacillus reuteri* [89]. The main active antimicrobial fraction of reuterin is acrolein. Acrolein is proposed to interfere with DNA synthesis by inhibiting the activity of bacterial ribonucleotide reductase [89,191]. Several studies found that reuterin and reuterin-related compounds exhibit antimicrobial and antibiofilm activities against wide range of Gram-positive and -negative bacteria including *B. subtilis, Clostridium difficile, E. coli, Fusobacterium nucleatum*, *Listeria* spp. and *P. aeruginosa* [67,89,191,192,193,194]. Reuterin also suppressed the growth of *Campylobacter* strains with MIC range between 1.5 to 5.8 μM. Use of bacteriocins and reuterin compounds appears to be a promising avenue to explore for control of campylobacters in poultry and poultry products by adding these natural products as feed additives in poultry diet [195].

Glycolipid Biosurfactant: Glycolipids, with potential anticancer and antimicrobial activities, already have a wide range of therapeutic applications including in pharmaceutical, food, and petroleum industries [196]. Sophorolipid is one of glycolipid molecules produced by the yeast *Starmerella bombicola* with antimicrobial and antibiofilm activities against foodborne pathogens such as *E. coli*, *Salmonella* spp. and *C. jejuni* [88,197,198]. Sophorolipid acts by inducing lysis of the cell membrane of pathogens, resulting in the release of cytoplasm contents [88,199]. Sophorolipid is a promising natural antimicrobial compound composed of biodegradable carbohydrate-based molecules with mild cytotoxicity, which makes them very attractive for the poultry industry [88,200]. One suggested application of sophorolipid is in the preservation and decontamination of meat products [88,200,201]. Silveira et al. [88], found that the combination of sophorolipid and lactic acid to treat *Campylobacters* cells resulted in an additive interaction and reduced the concentration required to treat campylobacters by 50%. Although lactic acid is commonly used in the poultry industry [202,203] and is approved by Food and Drug Administration (FDA), the treatment may negatively affect product quality by inducing changes in colour and flavour [204]. The combination of sophorolipid and lactic acid at reduced concentrations could provide an alternative treatment which would minimise the microbial contamination and preserve the aesthetic appeal and flavour of the foodstuffs.

## 4. Concluding Remarks

Solving the problem of gastroenteritis due to *C. jejuni* is an important challenge not only for improving the microbiological safety of food worldwide, but for reducing the enormous economic burden of hospitalisation, treatment, and loss of productivity caused by infection with these organisms. We postulate that the ability of *C. jejuni* to integrate into mixed-species biofilms is central for the efficient intestinal colonisation of the avian host and its transmission to human host. As is the case with other enteric pathogens, abolishing the ability to integrate into such a biofilm, will effectively limit transmission of this pathogen and reduce the incidence of disease in the human population. Therefore, innovative approaches of targeting zoonotic pathogens at the point of transmission from animal hosts to humans have an enormous potential to reduce or eliminate human infections, limiting the need for hospitalisation, treatment and vaccination.

Using natural products to disrupt the chain of pathogen transmission by using these as animal food additives, packaging disinfectants and bacterial growth inhibitors, offers great potential for an antibiotic-free path for foodstuffs from farm to fork. Furthermore, the application of natural compounds to enhance the efficacy of antibiotics, currently used to treat food-borne infections, offers additional advantages in our fight against the rise of antibiotic resistance. Further investigation of practical application of naturally occurring antibiofilm and antimicrobial compounds is required in order to progress the development of future preventative and therapeutic strategies to control the transmission of food borne diseases.

## Figures and Tables

**Table 1:** Antibiofilm activity of natural compounds with their mechanism of action.

Compounds		Mechanism of Action	Strains	MIC *	References
		Plant-derived compounds			
Essential oils (EOs)	- Cinnamaldehyde	- breakdown of the extracellular matrix - inhibit the activity of AI-2 molecules	*C. jejuni* NCTC 11168 *C. coli* *C. jejuni* S-8 *C. jejuni* NCTC 81-176 *C. jejuni* RC039	1.76 mg/L (75.64 mM)	[70,71]
	- Clove oil			0.05–0.4 mg/mL	[72]
	- Eugenol			2.69 mg/L (60.9 mM)	[73]
	- Carvacrol			31.25 mg/L (66.56 mM)	[74]
	- Lavender essential oil			1 mg/mL	[75]
	- Juniper essential oil			1 mg/mL	[74,76]
	- (-)-α-Pinene			125 mg/L	[77]
Plant extracts	- Grapefruit seed extract (GSE)	- break-down the outer membranes - inhibit the activity of AI-2 molecules	*C. jejuni* NCTC 11168 *C. jejuni* S-8 *C. jejuni* F38011 *C. jejuni* 180ip *C. jejuni* 238ip *C. coli*	60 mg/L	[78]
	- Citrus limon peel extract			225 µg/mL	[79]
	- Ethanol solution extract (EREE)			64–1024 µg/mL	[80]
	- Green tea (epigallocatechin gallate)			50 μg/mL	[81,82]
	- Polyphenolic extracts			0.15–0.3 mg/L	[83]
	- Resveratrol			0.1–0.2 mg/mL	[84]
	- Diallyl sulphide			0.04 mg/mL	[85]
Antimicrobial peptides (AMPs)	Puroindoline A (PinA)	- quorum sensing-mediated inhibition of EPS production.	*C. jejuni* 81-176	512 μg/mL	[56,86,87]
		Microorganism-derived compounds			
Algae extracts	*Delisea pulchra* extract	- inhibit the activity of AI-2 molecules	*C. jejuni* NCTC 11168	230 µg/mL	[88]
d-amino acids (DAs)	- d-Methionine - d-Tryptophan - d-Serine - d-Alanine	- consequence of incorporation of the DAs into the cell. - breakdown of the extracellular matrix such as EPS	*C. jejuni* NCTC 11168	5–100 mM	[47]
Probiotic-derived factors	- Bacteriocin - Reuterin	- interfering with DNA synthesis - interfering with the membrane integrity of bacterial cells	*C. jejuni* *C. coli*	0.025–32 µg/mL 1.5–5.8 μM	[89] [90]
Glycolipid Biosurfactant	Sophorolipid	- lysis of the cell membrane	*C. jejuni* subsp. *jejuni* 33560	0.003%	[88]

* Minimum inhibitory concentrations (MIC) as determined by the broth microdilution method described in individual references.

## Data Availability

Not applicable.
